# Analgosedation for catheter ablation in deep sedation: comparison between cryoballoon and pulsed field ablation

**DOI:** 10.1093/europace/euag126

**Published:** 2026-06-11

**Authors:** Moritz Nies, Laura Rottner, Ingrid Unruh, Julius Obergassel, Jan Rieß, Niklas Schenker, Djemail Ismaili, Maria Luisa Benesch Vidal, Dominik Christmann, Johanna Skibowski, Lea Scharlemann, Christoph Otto, Shinwan Kany, Marc D Lemoine, Bruno Reißmann, Feifan Ouyang, Paulus Kirchhof, Andreas Metzner, Andreas Rillig

**Affiliations:** Department of Cardiology, University Medical Center Hamburg-Eppendorf, Martinistraße 52, Hamburg 20246, Germany; German Center for Cardiovascular Research (DZHK), Partner Site North, Germany; Department of Cardiology, University Medical Center Hamburg-Eppendorf, Martinistraße 52, Hamburg 20246, Germany; Department of Cardiology, Universitäres Herz- und Gefässzentrum Frankfurt, Frankfurt, Germany; Department of Cardiology, University Medical Center Hamburg-Eppendorf, Martinistraße 52, Hamburg 20246, Germany; Department of Cardiology, University Medical Center Hamburg-Eppendorf, Martinistraße 52, Hamburg 20246, Germany; Department of Cardiology, University Medical Center Hamburg-Eppendorf, Martinistraße 52, Hamburg 20246, Germany; Department of Cardiology, University Medical Center Hamburg-Eppendorf, Martinistraße 52, Hamburg 20246, Germany; Department of Cardiology, University Medical Center Hamburg-Eppendorf, Martinistraße 52, Hamburg 20246, Germany; Department of Cardiology, University Medical Center Hamburg-Eppendorf, Martinistraße 52, Hamburg 20246, Germany; Department of Cardiology, University Medical Center Hamburg-Eppendorf, Martinistraße 52, Hamburg 20246, Germany; Department of Cardiology, University Medical Center Hamburg-Eppendorf, Martinistraße 52, Hamburg 20246, Germany; Department of Cardiology, University Medical Center Hamburg-Eppendorf, Martinistraße 52, Hamburg 20246, Germany; Department of Cardiology, University Medical Center Hamburg-Eppendorf, Martinistraße 52, Hamburg 20246, Germany; Department of Cardiology, University Medical Center Hamburg-Eppendorf, Martinistraße 52, Hamburg 20246, Germany; Department of Cardiology, University Medical Center Hamburg-Eppendorf, Martinistraße 52, Hamburg 20246, Germany; Department of Cardiology, University Medical Center Hamburg-Eppendorf, Martinistraße 52, Hamburg 20246, Germany; Department of Cardiology, University Medical Center Hamburg-Eppendorf, Martinistraße 52, Hamburg 20246, Germany; Department of Cardiology, University Medical Center Hamburg-Eppendorf, Martinistraße 52, Hamburg 20246, Germany; German Center for Cardiovascular Research (DZHK), Partner Site North, Germany; Department of Cardiology, University Medical Center Hamburg-Eppendorf, Martinistraße 52, Hamburg 20246, Germany; Department of Cardiology, University Medical Center Hamburg-Eppendorf, Martinistraße 52, Hamburg 20246, Germany

**Keywords:** Atrial fibrillation, Deep sedation, Analgesia, Pulsed field ablation, Pulmonary vein isolation

Pulsed field ablation (PFA) has been established as a viable alternative to thermal ablation for pulmonary vein isolation (PVI).^1–3^ Although general anaesthesia was recommended in early stages, deep sedation has been shown to be safe and feasible for the pentaspline (ps) PFA system.^4–6^ Skeletal muscle activation, phrenic nerve stimulation, and oesophageal contraction have caused discussions about sedation protocols for PFA in deep sedation.^7^ Data on the dose of sedation agents during PFA procedures as compared to cryoballoon ablation (CBA) are scarce and stem from small patient cohorts.^8^ This sub-analysis from a prospective, all-comer registry study aimed at comparing the use of sedation and analgesia medication and safety during index PVI using CBA and psPFA.

Patients undergoing index PVI in deep sedation were consecutively enrolled into TRUST, a prospective, single-centre, clinical cohort study (NCT05521451).^9^ Data of this study are available upon reasonable request. Ablation workflows for CBA and psPFA have been described before.^10^ No adjunctive ablation sets other than PVI were included for this analysis. Patients who underwent general anaesthesia (e.g. due to severe obesity with a body mass index ≥40 kg/m^2^, severe pulmonary comorbidity) were excluded. The ablation modality depended on system availability in the respective electrophysiology laboratories, irrespective of patient characteristics. Analgosedation was delivered by a centralized team consisting of cardiologists and specifically trained EP-nurses, operating uniformly across all electrophysiology laboratories. No standard premedication was administered before the procedure. Sedation was initiated using a bolus of 0.5 mg/kg propofol and 25 µg fentanyl and maintained via continuous propofol infusion at 6–7 mg/(kg*h). Additional boli or higher propofol rates were applied when sedation depth or analgesia were deemed insufficient (e.g. motoric reactions to PFA applications or increases in blood-pressure). Intraprocedural monitoring included continuous measurement of blood oxygen saturation, ECG, and non-invasive blood-pressure measurement at 5 min-intervals (or more frequently if needed). To account for procedure time and patient weight, propofol and fentanyl doses were indexed to both parameters (mg/(kg*min); ng/(kg*min)). All analyses were exploratory using appropriate univariate testing. Two-sided *P*-values <0.05 were considered statistically significant. All statistical analyses were performed in SPSS 30.0 (IBM Corp., Armonk, NY).

In total, 400 procedures were analyzed (200 CBA, 200 PFA). After excluding procedures with different PFA systems or incomplete datasets, 375 patients (192 CBA, 183 psPFA) were included. There were no differences in baseline characteristics between both groups other than a higher rate of paroxysmal AF in the psPFA-group (50.3% vs. 34.4%; *P* = 0.006). All procedures were completed successfully. To account for an adaptation phase with the new device, the initial 50 patients of each group were analyzed separately for procedural data analyses. After the adaptation phase, fentanyl requirement was significantly higher with psPFA than with CBA (6.17 [IQR 4.46–10.02] vs. 5.74 [IQR 4.05–7.69] ng/(kg*min), *P* = 0.04, *Figure 1A and C*). Propofol use was similar in both groups (0.14 [IQR 0.11–0.17] vs. 0.13 [IQR 0.11–0.17] mg/(kg*min); *P* = 0.62; *Figure 1A and B*). No differences in medication dose were observed in the initial 50 patients. Sedation-related adverse events were rare and did not differ significantly between CBA and psPFA: Intraprocedural pulmonary aspiration occurred in 2/183 patients (1.1%) with psPFA, one of which required intermittent bag ventilation (1/183; 0.5%). Both patients received empirical antibiotic therapy to prevent aspiration-related pneumonia and were discharged without prolonged hospitalization. Unplanned vasopressor use occurred in 1/183 patient (0.5%) with psPFA with intermittent hypotension during suspected right coronary artery air embolism. Coronary angiography showed no stenoses, and the patient fully recovered without intervention. This complication was considered unrelated to sedation. No sedation-related complications occurred with CBA. No conversion to general anaesthesia was needed. Fluoroscopy time and dose were higher in the PFA group (10.9 [IQR 7.6–13.9] vs. 9.4 [IQR 6.5–13.5] min; *P* = 0.016 and 372.7 [IQR 248.1–549.3] vs. 309.5 [IQR 205.7–559.1]; *P* = 0.002, respectively). PsPFA procedures were significantly longer than CBA procedures for the initial 50 patients (86 [IQR 68–107] min vs. 68 [54–82] min, *P* < 0.001) but significantly shorter than CBA procedures after the adaptation phase (54 [IQR 37–72] min vs. 60 [IQR 50–70] min, *P* = 0.027).

**Figure 1:**
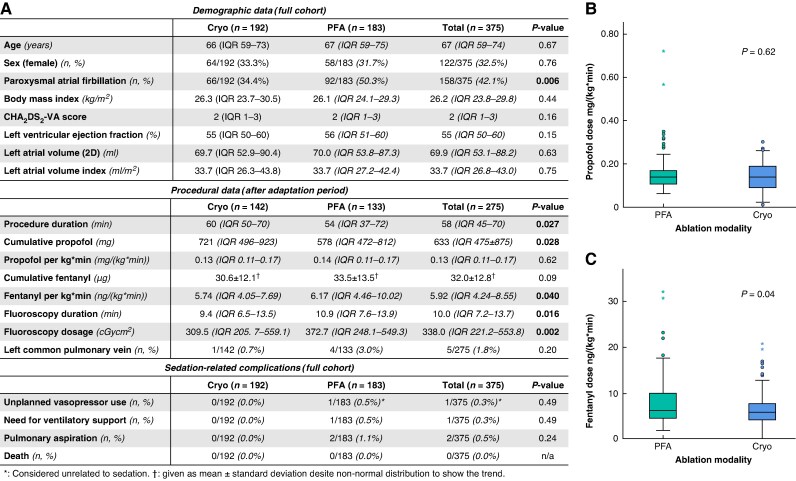
Demographic, procedural, and safety data. (*A*): Table showing demographic data (top), procedural parameters (middle), and sedation-related complications (bottom). A higher cumulative propofol dose was observed for CBA procedures, which was driven by longer procedure times. When adjusted for procedure time and patient weight, propofol use was similar in both groups. (*B*): propofol and (*C*): fentanyl doses for pulsed field and cryoballoon ablation are visualized as box plots. Boxes illustrate median and IQR, dots show extreme values. PFA, pulsed field ablation.

Overall, the findings demonstrate a higher fentanyl requirement in psPFA procedures, which may be related to procedure-associated skeletal muscle and nerve activation. This poses an additional challenge for sedation as compared to CBA procedures. A prior study reported higher propofol, midazolam, and sufentanyl use in PFA procedures. Notably, the most pronounced difference was observed for sufentanyl.^8^ Although a different sedation protocol was used in this study, our results confirm the increased analgesia requirement for the use of fentanyl. Overall safety of deep sedation was satisfactory for both ablation modalities. Propofol doses were similar with both energy sources, potentially due to the standardized dosing protocol in our centre. Of note, both safety events represented intraprocedural pulmonary aspirations in the psPFA-group. This is in line with a prior study, in which the only sedation-related complication was pulmonary aspiration during a psPFA procedure.^8^ PFA-induced phrenic nerve stimulation or oesophageal contraction could contribute to pulmonary aspiration, especially when combined with higher opioid doses in deep sedation. This analysis suggests that such events are rare and does not establish a link to a specific energy source. The longer procedure times in the first 50 psPFA cases are likely explained by a learning curve with the novel device. Additionally, three-dimensional remapping was performed in selected early psPFA cases to understand lesion size. After the adaptation phase, procedure times for psPFA were faster as has been shown consistently in recent studies. The higher fluoroscopy time and dose for psPFA might be due to mandatory catheter repositioning and lack of mapping integration.

While this is one of the largest analyses comparing analgesia and sedative dosing between CBA and PFA procedures, it is primarily limited by its observational design and the lack of a standardized, blinded analysis approach.

In summary, both psPFA and CBA appear safe and effective in deep sedation. Significantly higher opioid doses during psPFA procedures suggest an increased need for analgesia to mitigate pain from energy delivery, skeletal muscle activation, or nerve stimulation. Monitoring for signs of pulmonary aspiration during PFA procedures could further reduce sedation-related risks.

## References

[1] Turagam MK, Aryana A, Day JD, Dukkipati SR, Hounshell T, Nair D et al Multicenter study on the safety of pulsed field ablation in over 40,000 patients: MANIFEST-US. J Am Coll Cardiol 2026;87:172–93. 41389071 10.1016/j.jacc.2025.10.051

[2] Tzeis S, Gerstenfeld EP, Kalman J, Saad EB, Sepehri Shamloo A, Andrade JG et al 2024 European heart rhythm association/heart rhythm society/Asia Pacific heart rhythm society/Latin American heart rhythm society expert consensus statement on catheter and surgical ablation of atrial fibrillation. Europace 2024;26:euae043.38587017

[3] Chun KJ, Miklavcic D, Vlachos K, Bordignon S, Scherr D, Jais P et al State-of-the-art pulsed field ablation for cardiac arrhythmias: ongoing evolution and future perspective. Europace 2024;26:euae134. 38848447 10.1093/europace/euae134 PMC11160504

[4] Schmidt B, Bordignon S, Tohoku S, Chen S, Bologna F, Urbanek L et al 5S study: safe and simple single shot pulmonary vein isolation with pulsed field ablation using sedation. Circ Arrhythm Electrophysiol 2022;15:e010817. 35617232 10.1161/CIRCEP.121.010817

[5] Rillig A, Hirokami J, Moser F, Bordignon S, Rottner L, Shota T et al General anaesthesia and deep sedation for monopolar pulsed field ablation using a lattice-tip catheter combined with a novel three-dimensional mapping system. Europace 2024;26:euae270. 39576055 10.1093/europace/euae270 PMC11583048

[6] Chun KRJ, Plank K, Neven K, Reichlin T, Blaauw Y, Hansen J et al Characterization of sedation strategies in real-world use of pulsed field ablation sub-analysis of the EU-PORIA registry. Europace 2025:euaf287. 41248987 10.1093/europace/euaf287

[7] Nies M, Koruth JS, Mlcek M, Petru J, Tibenská VC, Watanabe K et al Is the esophagus spared during pulsed field ablation? Early histopathology and in vivo esophageal retraction. Heart Rhythm 2025;23:788–99. 40581235 10.1016/j.hrthm.2025.06.033

[8] Wahedi R, Willems S, Feldhege J, Jularic M, Hartmann J, Anwar O et al Pulsed-field versus cryoballoon ablation for atrial fibrillation-impact of energy source on sedation and analgesia requirement. J Cardiovasc Electrophysiol 2024;35:162–70. 38009545 10.1111/jce.16141

[9] Obergassel J, Rieß JL, Jaeckle S, van Elferen S, Nies M, Schenker N et al Long-term outcome and predictors for recurrence after medical and interventional treatment of arrhythmias at the UniverSity heart CenTer Hamburg (TRUST): design and patient profile snapshot of a prospective clinical cohort study (accepted manuscript). Eur Heart J Open 2026;6:oeag002. 41743284 10.1093/ehjopen/oeag002 PMC12930198

[10] Lemoine MD, Obergassel J, Jaeckle S, Nies M, Taraba S, Mencke C et al Pulsed-field- vs. Cryoballoon-based pulmonary vein isolation: lessons from repeat procedures. Europace 2024;26:euae221. 39166530 10.1093/europace/euae221 PMC11363872

